# Iron Availability for Micro-Tom Tomatoes via Nanoparticles: Implications for the Antioxidant System and Carbon Metabolism

**DOI:** 10.3390/plants15193033

**Published:** 2026-10-04

**Authors:** Rafaela Hemily Cirilo, João Pedro Sampaio Gama, Felipe Girotto Campos, Carla dos Santos Riccardi, Carmen Sílvia Fernandes Boaro

**Affiliations:** 1 Department of Biodiversity and Biostatistics, Institute of Biosciences, São Paulo State University—UNESP, Botucatu 18618-689, Brazil; rafaela.cirilo@unesp.br (R.H.C.); joao.gama@unesp.br (J.P.S.G.); carmen.boaro@unesp.br (C.S.F.B.); 2 Department of Bioprocesses and Biotechnology, School of Agricultural Sciences, São Paulo State University—UNESP, Botucatu 18610-034, Brazil; carla.riccardi@unesp.br

**Keywords:** photosynthesis, oxidative stress, dry mass production, hydrogen peroxide

## Abstract

*Solanum lycopersicum* converts Fe^3+^ into Fe^2+^, the form absorbed by plants. The Fe^2+^ present in the growth medium can be absorbed in excess by the plants, causing phytotoxicity. Magnetite has an isocratic balance of Fe^3+^/Fe^2+^, which enables the release of Fe^2+^ in the culture medium. The literature reports that high availability of Fe^2+^ can cause oxidative damage and a reduction in dry mass, whereas the application of maghemite nanoparticles, which release only Fe^3+^, can promote an increase in biomass. Thus, we investigated the influence of iron oxides (magnetite and maghemite) on photosynthesis, leaf carbohydrate profile and the enzymatic antioxidant system of *Solanum lycopersicum* “cv Micro-Tom”. Our results indicated that application of iron oxide nanoparticles provided sufficient iron to reverse the element’s deficiency, restoring the photochemical apparatus. However, the supply of magnetite resulted in reduced root dry mass, an accumulation of reducing sugars, and increased antioxidant enzyme activity. Maghemite may have contributed to a greater allocation of photoassimilates to reproductive organs, such as flowers, which showed a higher percentage distribution of dry mass, indicating its potential for use in plant cultivation.

## 1. Introduction

Plants require an adequate supply of iron (Fe) for their development. Excessive Fe uptake can result in toxicity, whereas insufficient Fe uptake causes deficiency, described in several crop plants, such as rice, soybean and sorghum in regions of the Cerrado [1]. In addition to the availability of Fe in the soil, other factors that impact the imbalance of this micronutrient in plants are its absorption strategy and the solubility of this element in the soil.

Chelated fertilizers are characterized by rapid availability of Fe, and the demand for recurrent applications in the soil increases the cost of production [2]. However, the application of chelated Fe nanoparticles allows for a slow availability of the element, reducing the need for recurrent applications [3]. For instance, the use of iron oxide nanoparticles (NPs) has become attractive in agriculture for ecological and efficient use [3], contributing to tackling the effects of food shortages, heavy metal contamination and other abiotic pressures [4], such as Fe_3_O_4_, which can act as a nanopesticide, preventing diseases in plants by increasing their immunity [5]. Some nanomaterials can be biosynthesized as sustainable alternatives to conventional chemical fertilizers [6].

Nanoparticles are defined as nanoscale particles with at least one of their external dimensions ranging from 1 to 100 nm [7]. Dietz and Herth [8] refer to them as a class of materials, with a high tendency for aggregation, synthesized through biotic or abiotic chemical processes, or by combustion.

Within plant tissues, the movement of nanoparticles occurs via two pathways. The symplastic route involves passing through the membrane, while the apoplastic route is facilitated by the size of the pores present in the cell wall. In the cells of the roots and the palisade parenchyma, the diameter of these pores varies, respectively, between 3.5 and 3.8 nm and 4.5–4.8 nm. In this sense, only nanoparticles with diameters smaller than 5 nm are able to cross these pores without causing cell damage [8,9].

Due to accumulation and transport, iron NPs are able to transfer electrons to O_2_, generating reactive oxygen species (ROS), which are responsible for cell membrane lipid peroxidation and phytotoxicity in plants. In response, there is activation of antioxidant enzymes, such as superoxide dismutase (SOD), which performs the dismutation of superoxide resulting from photorespiratory processes and the glycolate and catalase (CAT) and peroxidase (POD) pathways, which act in the detoxification of hydrogen peroxide (H_2_O_2_), transforming it into water and O_2_ [8,10]. Changes in the cell wall of iron-deficient cells are closely related to low POD activity [11], which impairs lignin biosynthesis, leading to structural cell damage in the epidermis and rhizoderm [12]. Caramanico et al. [13] refer to the importance of iron as a cofactor for the functioning of antioxidant enzymes, and the lack of the element can result in extensive structural damage and lipid peroxidation.

Depending on the concentration, iron nanoparticles deliver the ion and have low or no cytotoxicity, DNA damage, and oxidative stress [14,15]. They reduce stress from heavy metals, as they present adsorption efficiency for these metals, contributing to the elimination of their stressful effects on vegetables [5].

High concentrations of iron nanoparticles are harmful to germination, as in the study of El-Temsah et al. [16], where inhibitory effects on germination were observed in aqueous suspensions with concentrations higher than 250 mg L^−1^ of iron nanoparticles. However, Kim et al. [17] report activation of the ATPase proton pump with a decrease in apoplastic pH when *Arabidopsis thaliana* is exposed to iron nanoparticles, causing the stomata to open. In addition, the authors observed, under these conditions, an increase in leaf area, which may contribute to greater light uptake and photosynthetic activity.

Magnetite (Fe_3_O_4_) and maghemite (γ-Fe_2_O_3_) are the main classes of iron oxide nanoparticles (NPs), which have become an important source of this metal for plant nutrition. The effects of iron oxide NPs in the soil are complex, and they can contribute to remediation by removing the contaminants present [5]. Depending on concentration, these NPs can act either as sources of Fe or as a phytotoxic agent [8].

Magnetite in wheat seedlings decreased heavy metal-induced oxidative stress by regulating the activity of the enzymes superoxide dismutase (SOD) and peroxidase (POD) in order to decrease reactive oxygen species (ROS) [18].

Magnetite features an isocratic balance of Fe^3+^/Fe^2+^ [19], which enables the release of Fe^2+^ in the culture medium. A concentration equal to 500 mg L^−1^ of Fe^2+^ in the cultivation of *Oryza sativa* L. × *Oryza glaberrima* Steud. promoted oxidative damage and reduction in dry mass [20]. However, the cultivation of micro-tom tomatoes showed satisfactory results when the plants were cultivated with 500 mg L^−1^ of magnetite [21]. The literature shows that plant species present different results when they are cultivated with different concentrations of magnetite, which may explain the better performance of plants supplied with 500 mg ^L−1^ of magnetite, corresponding to 250 mg L^−1^ Fe^3+^ and 250 mg L^−1^ Fe^2+^, compared with the 500 mg L^−1^ Fe^2+^ treatment that caused toxicity in rice.

Yousaf et al. and Rui et al. [22,23] reported root growth and increased biomass in seedlings of *Arachis hypogaea* and *Zea mays* treated with maghemite nanoparticles (γ-Fe_2_O_3_), indicating an increase in the partitioning of photoassimilates from leaves (source) to roots (drains). There was no toxic effect on *Chlorella* sp. and *Daphnia magna*, two aquatic species, exposed to Fe_2_O_3_ NPs [24].

Some studies reveal that iron oxide nanoparticles in the environment have the potential for toxicity depending on surface coating, concentration, size, and exposure duration, which can lead to bioaccumulation in organs [25]. However, other studies report that magnetite and maghemite nanoparticles exhibit low toxicity within organisms and are even used in clinical procedures [26,27]. These results were confirmed in *Oreochromis niloticus* fish exposed to magnetite and maghemite, as no tissue lesions were observed, indicating the absence or low toxicity of the nanoparticles [28]. It should be considered that an excess of available Fe^2+^ in the environment is likely to cause greater physiological alterations compared to conditions in which this element is associated with iron oxide nanoparticles [20,29,30].

Therefore, understanding the impact of Fe toxicity on plants, aggravated by anthropogenic action, has become a current issue that involves concern with iron metabolism in the plant, especially when its supply in the environment is excessive. Under these conditions, iron oxide NP fertilizers of different classes and concentrations may represent a promising alternative. In this study, we investigated the influence of magnetite and maghemite NPs on photosynthesis, leaf carbohydrate profile and the enzymatic antioxidant system of *Solanum lycopersicum* “cv Micro-Tom”.

## 2. Results

### 2.1. Chlorophyll a Fluorescence and Gas Exchange

**Figure 1:**
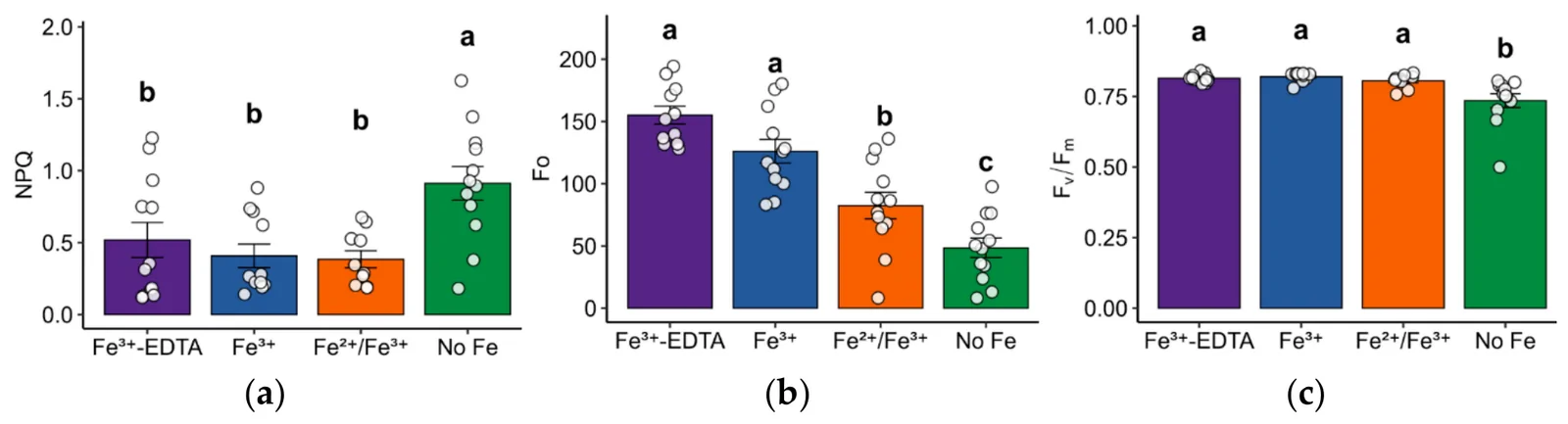
Chlorophyll *a* fluorescence: (**a**) non-photochemical quenching (NPQ); (**b**) minimum efficiency adapted to the dark (F0); (**c**) maximum potential quantum efficiency of PSII (Fv/Fm), in Micro-Tom tomato grown under different treatments. Control (Fe^3+^-EDTA); 125 mg L^−1^ of maghemite (Fe^3+^); 125 mg L^−1^ magnetite (Fe^2+^/Fe^3+^); No Fe. Equal letters mean that there was no significant difference (*p* ≥ 0.5, Mixed Model); bars represent the average per treatment, the points are the individual values of each plant per treatment, and the lines on the bars refer to the standard error.

Non-photochemical quenching (NPQ) and maximum quantum potential efficiency of PSII (Fv/Fm) did not differ between treatments with iron supply (Figure 1a,c). On the other hand, the minimum efficiency adapted to the dark (F_0_) was higher in plants subjected to the negative control and maghemite (Figure 1b).

**Figure 2:**
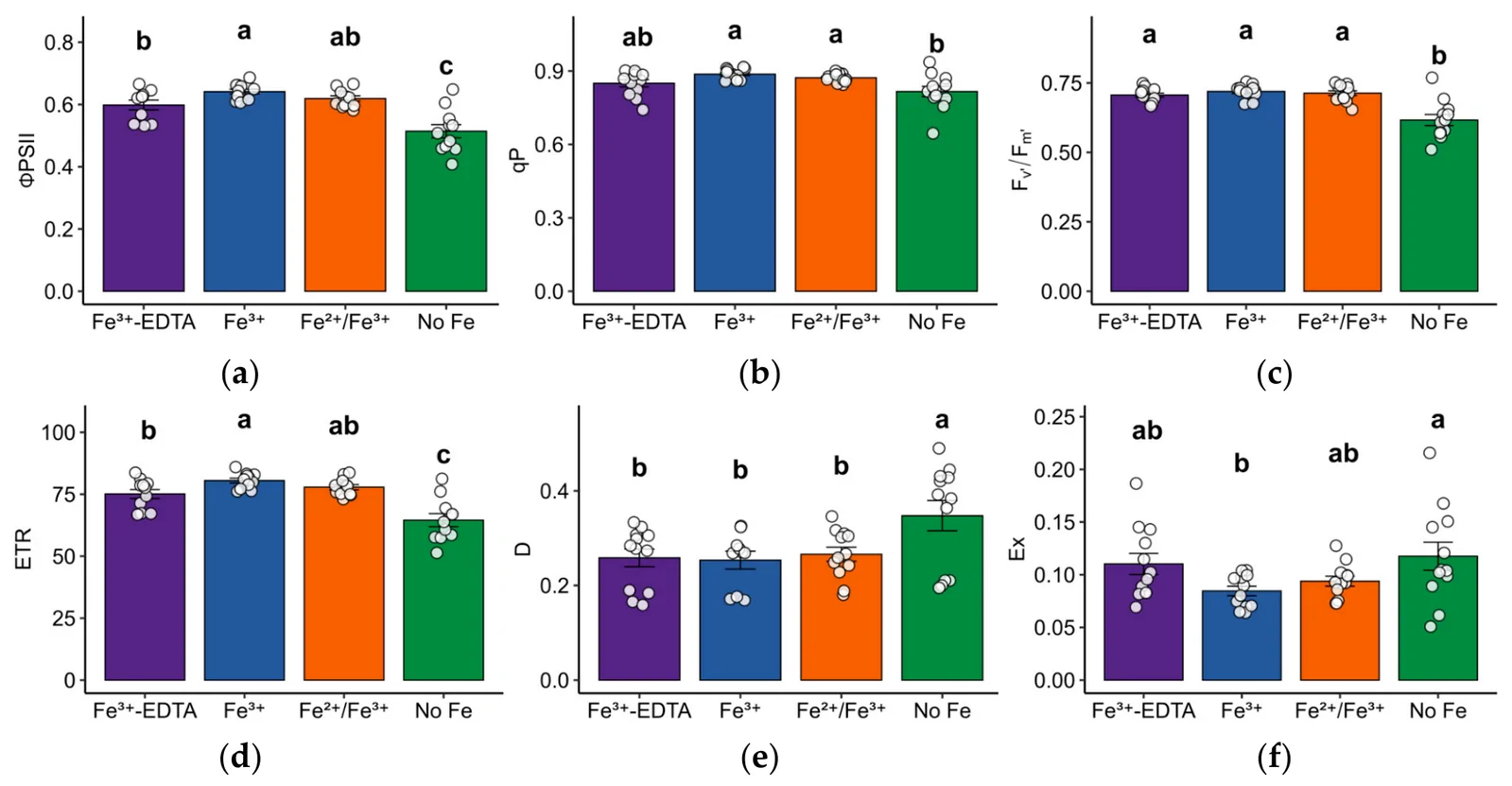
(**a**) Effective quantum yield of PSII (ΦPSII); (**b**) photochemical quenching (qP); (**c**) potential quantum efficiency of PSII (Fv′/Fm′); (**d**) electron transport rate (ETR); (**e**) heat dissipation in the antenna complex (D); (**f**) excess energy not dissipated or used in the photochemical phase (Ex), in the Micro-Tom tomato grown under different treatments. Control (Fe^3+^-EDTA); 125 mg L^−1^ of maghemite (Fe^3+^); 125 mg L^−1^ magnetite (Fe^2+^/Fe^3+^); No Fe. Equal letters mean that there was no significant difference (*p* ≥ 0.5, Mixed Model); bars represent the average per treatment, the points are the individual values of each plant per treatment, and the lines on the bars refer to the standard error.

There was a higher effective quantum yield of photosystem II (ΦPSII) in plants grown with maghemite compared to the control (Figure 2a). Regarding qP and Fv′/Fm′, the treatments with the nanoparticles did not differ from each other (Figure 2b,c). Plants subjected to maghemite showed higher ETR compared to the positive control (Figure 2d). There was greater heat dissipation (D) in plants grown without iron (Figure 2e). Plants subjected to maghemite showed lower accumulated energy (Ex) compared to those cultivated without iron (Figure 2f).

**Figure 3:**
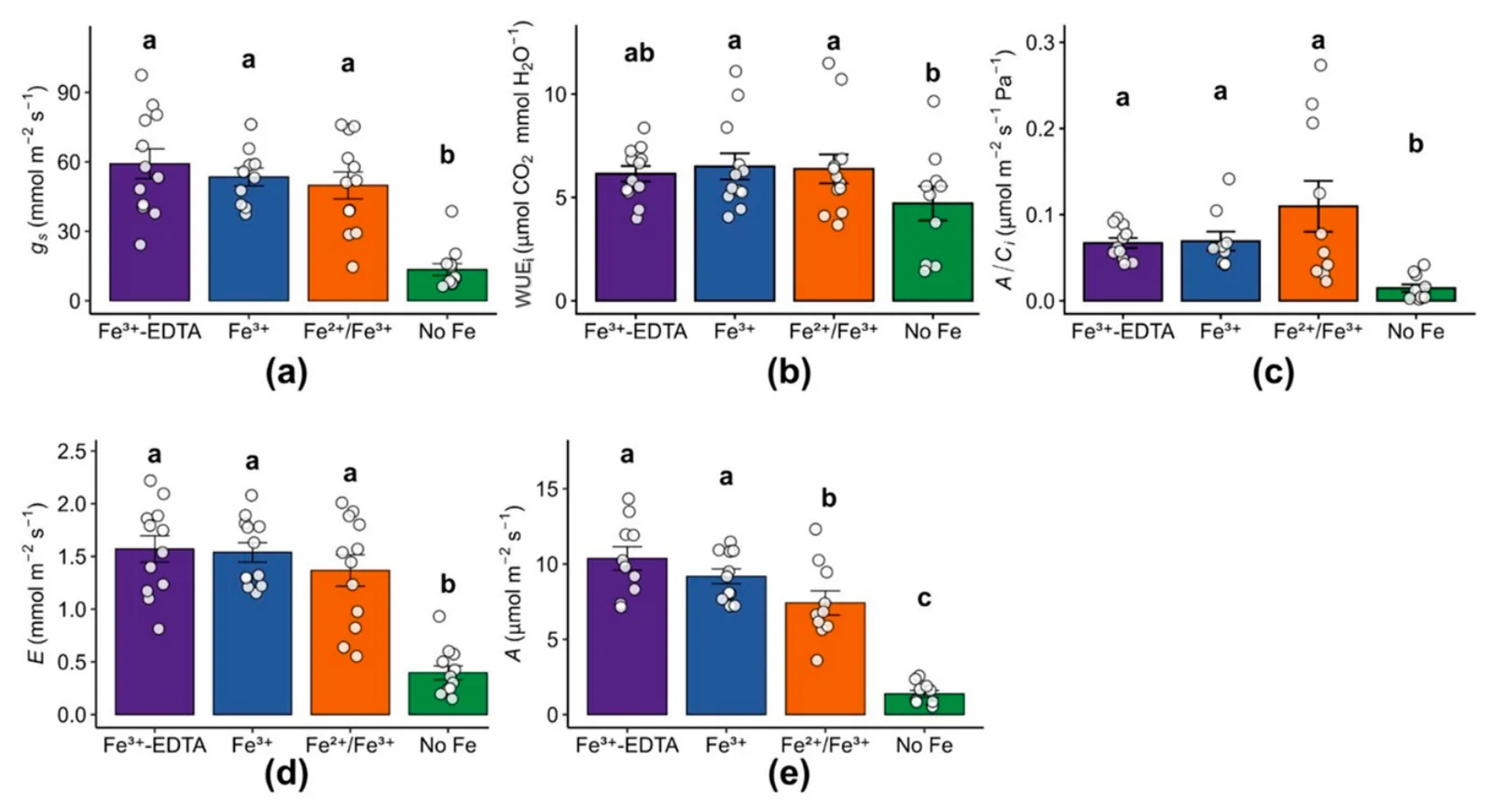
Gas exchange: (**a**) stomatal conductance (*g_s_*); (**b**) Instantaneous water use efficiency (WUEi); (**c**) instantaneous carboxylation efficiency (*A/C_i_*); (**d**) transpiration rate (*E*); (**e**) carbon assimilation rate (*A*) in the Micro-Tom tomato grown in the different treatments. Control (Fe^3+^-EDTA); 125 mg L^−1^ of maghemite (Fe^3+^); 125 mg L^−1^ magnetite (Fe^2+^/Fe^3+^); No Fe. Equal letters mean that there was no significant difference (*p* ≥ 0.5, Mixed Model); bars represent the average per treatment, the points are the individual values of each plant per treatment, and the lines on the bars refer to the standard error.

Considering gas exchange, plants cultivated with iron had no difference in stomatal conductance (*g_s_*), instantaneous carboxylation efficiency (*A/C_i_*) and transpiration rate (*E*) e (Figure 3a,c,d). Instantaneous water use efficiency (WUEi) did not differ in plants grown with nanoparticles; however, it was lower in those grown without iron (Figure 3b). There was lower carbon (*A*) assimilation in plants with magnetite compared to those with Iron-EDTA and maghemite (Figure 3e).

### 2.2. Photosynthetic Pigments

**Figure 4:**
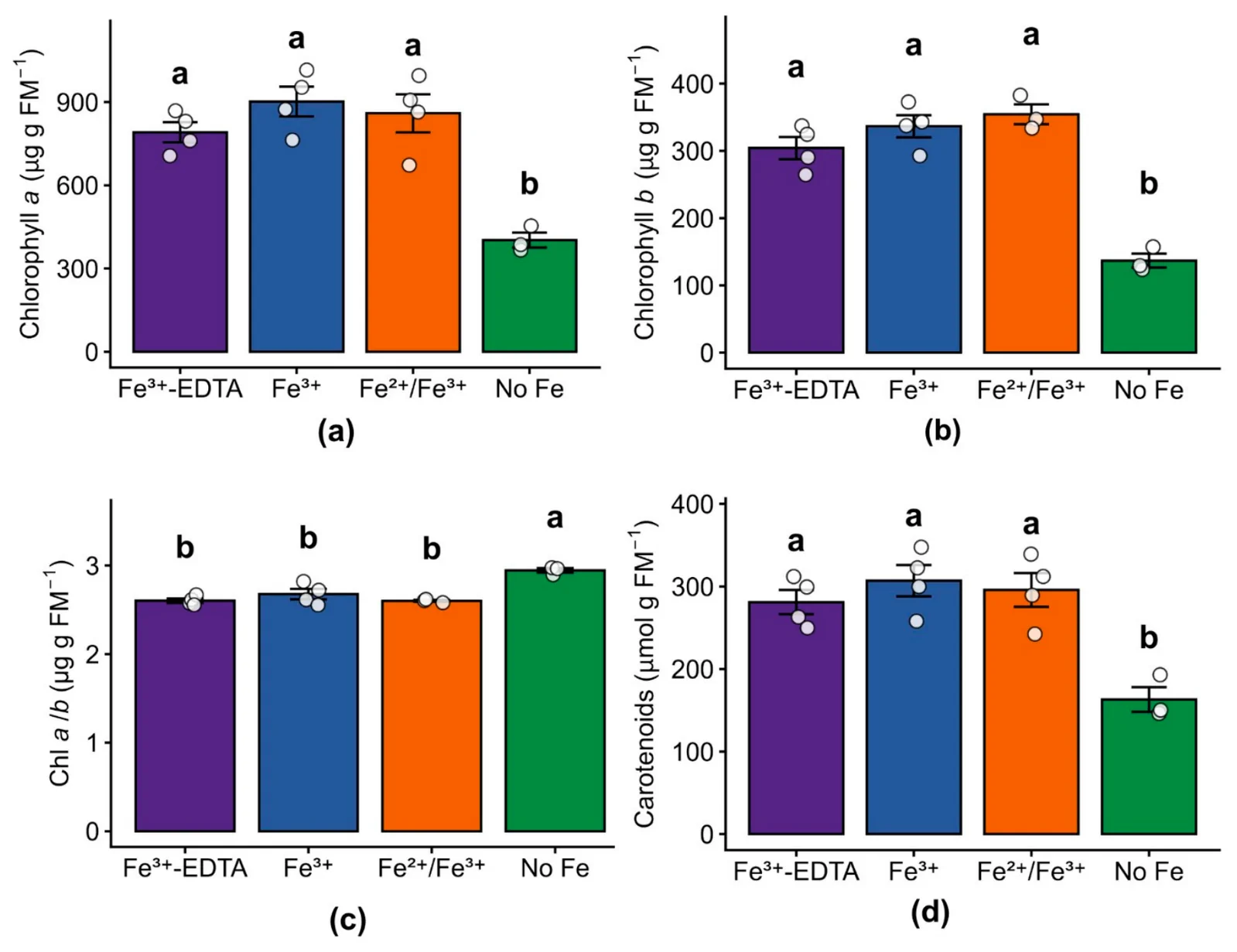
Photosynthetic pigments (μg pigment g fresh mass^−1^): (**a**) chlorophyll *a*; (**b**) chlorophyll *b*; (**c**) chlorophyll *a*/chlorophyll *b* ratio (Chl *a*/*b*); (**d**) carotenoids, in the Micro-Tom tomato grown in the different treatments. Control (Fe^3+^-EDTA); 125 mg L^−1^ of maghemite (Fe^3+^); 125 mg L^−1^ magnetite (Fe^2+^/Fe^3+^); No Fe. Equal letters mean that there was no significant difference (*p* ≥ 0.5, Generalized linear model); bars represent the average per treatment; the points are the individual values of each plant per treatment; and the lines on the bars refer to the standard error.

There was no difference in photosynthetic pigment content among plants supplied with iron, regardless of source (Figure 4a–d).

### 2.3. Soluble Carbohydrate Profile

**Figure 5:**
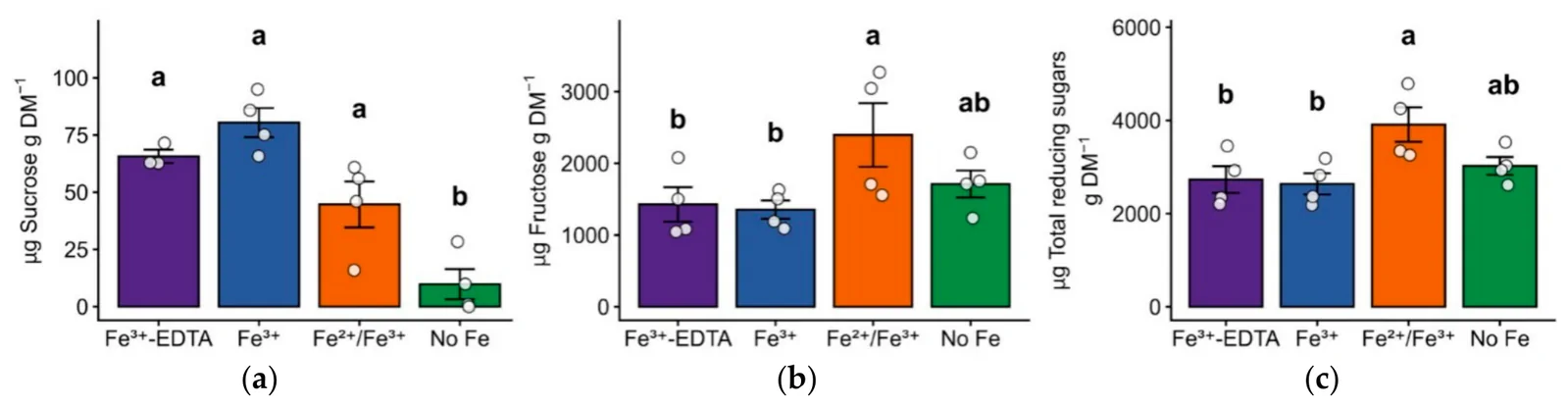
Soluble carbohydrate profile (μg sugar g dry mass^−1^): (**a**) sucrose; (**b**) fructose; (**c**) total reducing sugars, in Micro-Tom tomatoes grown in the different treatments. Control (Fe^3+^-EDTA); 125 mg L^−1^ of maghemite (Fe^3+^); 125 mg L^−1^ magnetite (Fe^2+^/Fe^3+^); No Fe. Equal letters mean that there was no significant difference (*p* ≥ 0.5, Generalized linear model); bars represent the average per treatment, the points are the individual values of each plant per treatment, and the lines on the bars refer to the standard error.

Iron treatments showed similarity in sucrose concentration (Figure 5a). However, there was a lower concentration of fructose and total reducing sugars (glucose, fructose and mannose) in plants grown in the presence of maghemite compared to those subjected to magnetite (Figure 5b,c).

### 2.4. Antioxidant System

**Figure 6:**
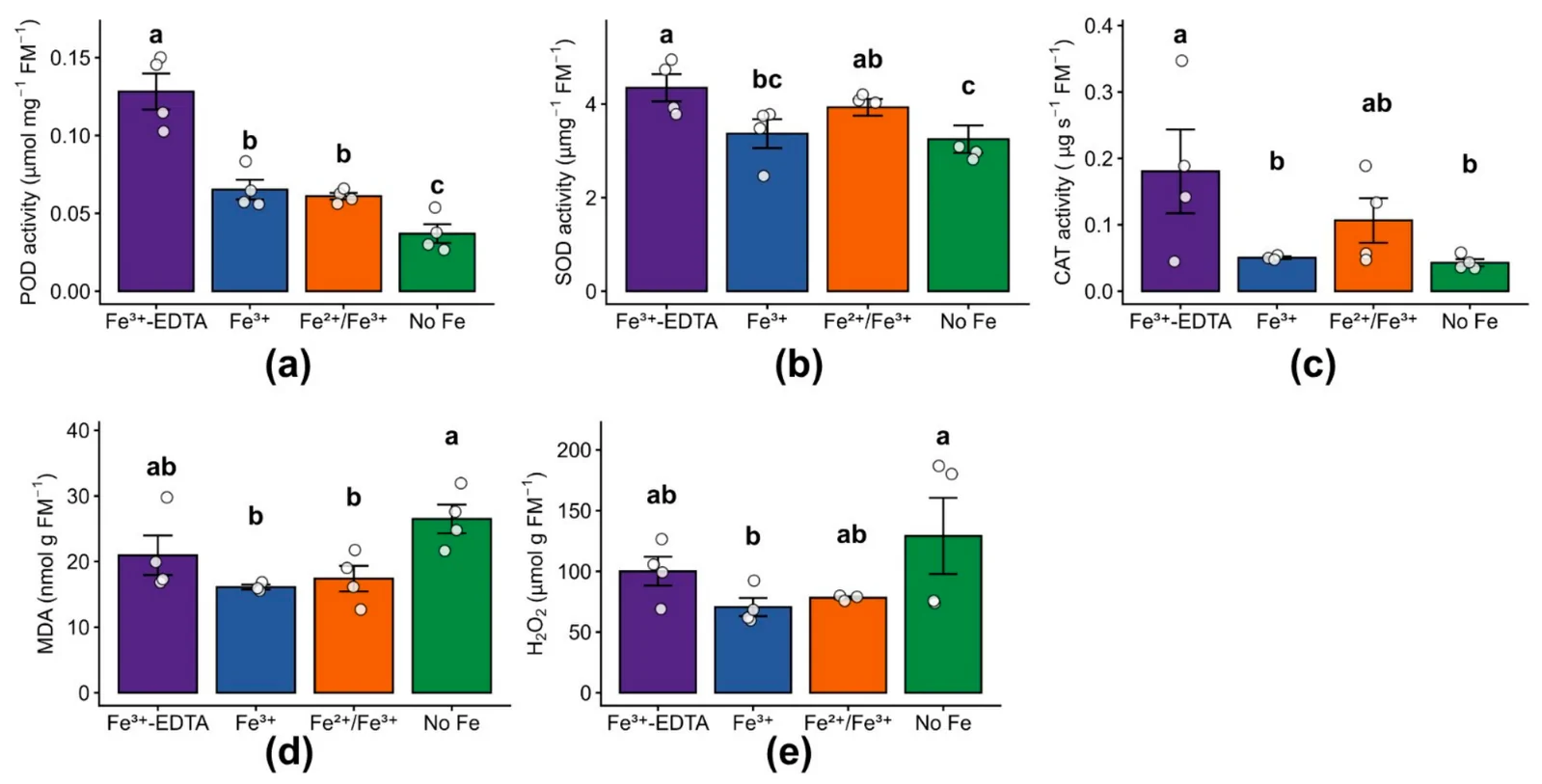
Antioxidant system: (**a**) peroxide (POD); (**b**) superoxide dismutase (SOD); (**c**) catalase (CAT); (**d**) lipid peroxidation (MDA); (**e**) hydrogen peroxide (H_2_O_2_), in the Micro-Tom tomato grown under different treatments: Control (Fe^3+^-EDTA); 125 mg L^−1^ of maghemite (Fe^3+^); 125 mg L^−1^ magnetite (Fe^2+^/Fe^3+^); No Fe. Equal letters mean that there was no significant difference (*p* ≥ 0.5, Generalized linear model); bars represent the average per treatment; the points are the individual values of each plant per treatment; and the lines on the bars refer to the standard error.

There was no difference in POD activity between the plants of the nanoparticle treatments, which were lower than the activity of those of the positive control, iron-EDTA (Figure 6a). There was no difference in the activity of SOD; there was similarity between the plants of the treatments with nanoparticles; however, those with maghemite showed lower activity in relation to iron-EDTA (Figure 6b). The enzyme catalase (CAT) showed lower activity in plants subjected to maghemite and absence of iron (Figure 6c). Plants grown with nanoparticles showed lower concentrations of malondialdehyde (MDA) than those without iron (Figure 6d), and those cultivated with maghemite had lower concentrations of H_2_O_2_ than those without iron (Figure 6e).

### 2.5. Dry Masses and Assimilate Partition

**Figure 7:**
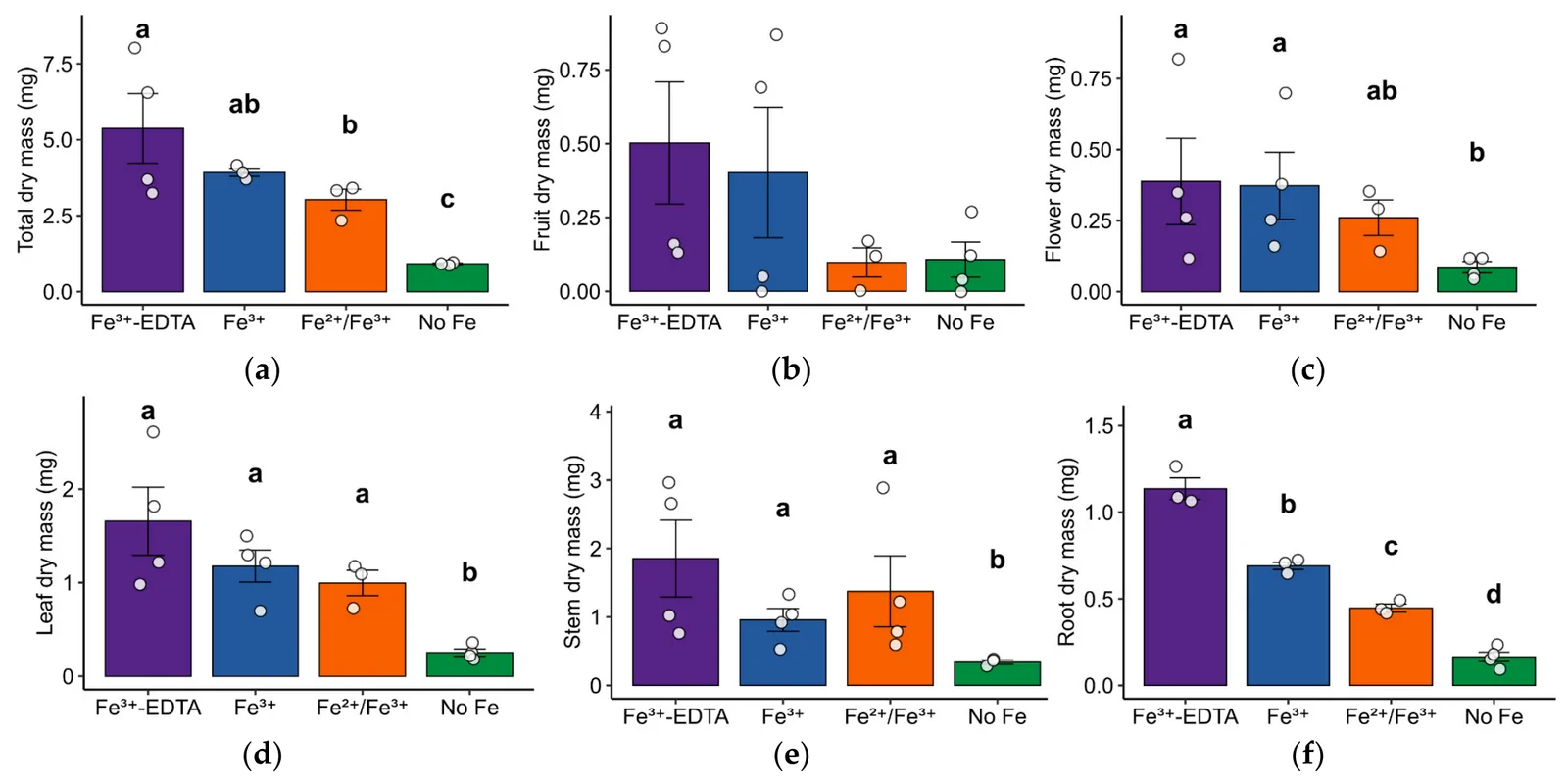
Dry mass in milligrams (mg) of Micro-Tom tomato grown under different treatments: (**a**) total dry mass; (**b**) dry mass of the fruit; (**c**) dry mass of the flower; (**d**) dry mass of the leaves; (**e**) dry mass of the stem; (**f**) dry mass of the roots. Control (Fe^3+^-EDTA); 125 mg L^−1^ of maghemite (Fe^3+^); 125 mg L^−1^ magnetite (Fe^2+^/Fe^3+^); No Fe. Equal letters mean that there was no significant difference (*p* ≥ 0.5, Generalized linear model); bars represent the average per treatment, the points are the individual values of each plant per treatment, and the lines on the bars refer to the standard error.

The total dry mass of plants cultivated with magnetite was lower than that of plants subjected to the positive control and similar to that of plants cultivated with maghemite (Figure 7a). However, there was no difference in fruit dry mass between the treatments (Figure 7b). Iron treatments did not differ in terms of dry mass of flowers, leaves and stems (Figure 7c–e). Regarding the roots, plants subjected to maghemite showed higher dry mass than plants cultivated with magnetite and without iron (Figure 7f).

**Figure 8:**
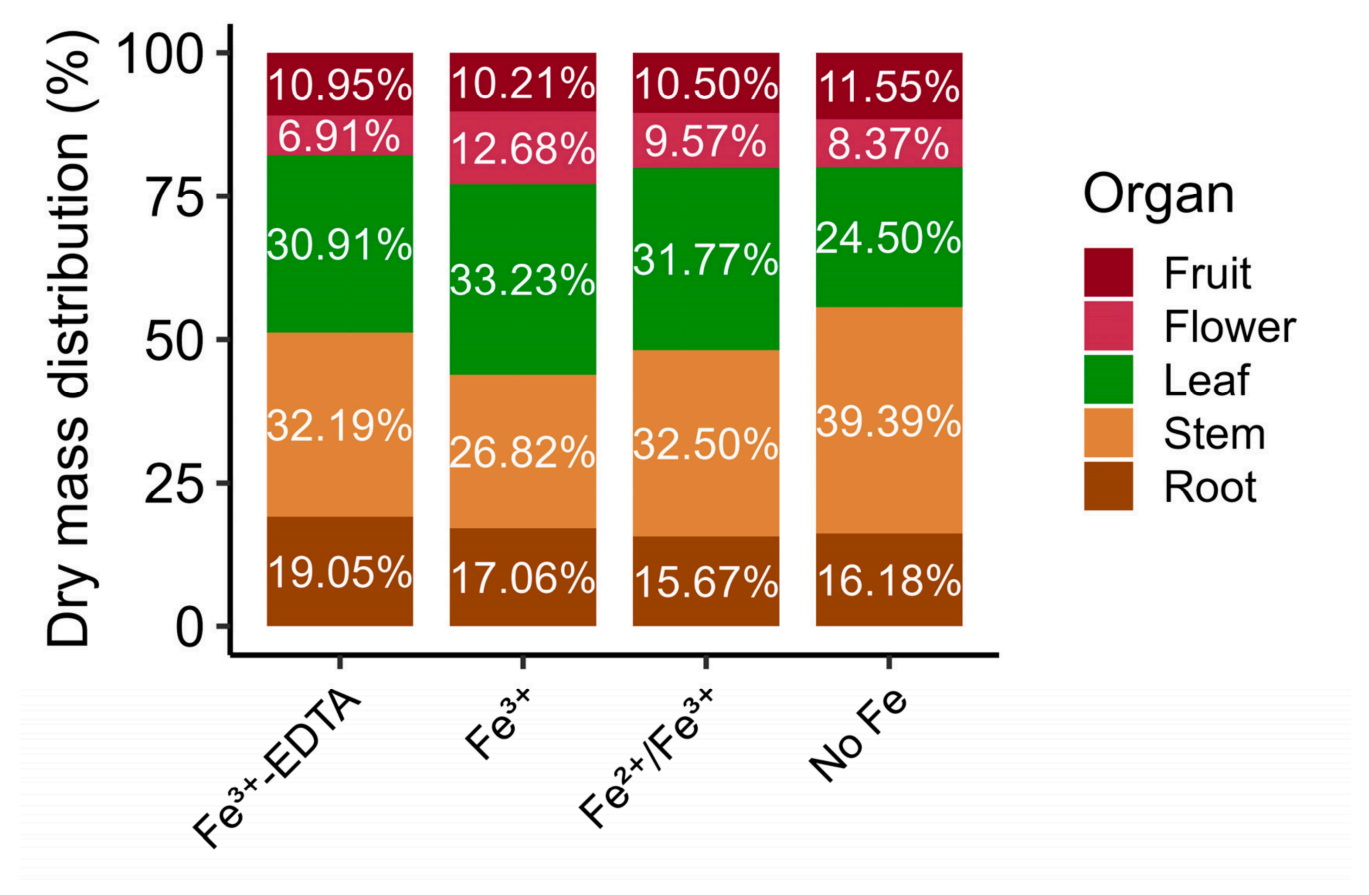
Dry mass partitioning by plant organ in Micro-Tom tomato grown in the different treatments: Fe^3+^-EDTA (control); Fe^3+^ (Maghemite); Fe^2+^/Fe^3+^ (Magnetite); No Fe (Iron-free treatment). Percentages in the bars represent the partition of dry mass for the different organs in relation to the total dry mass.

**Figure 9:**
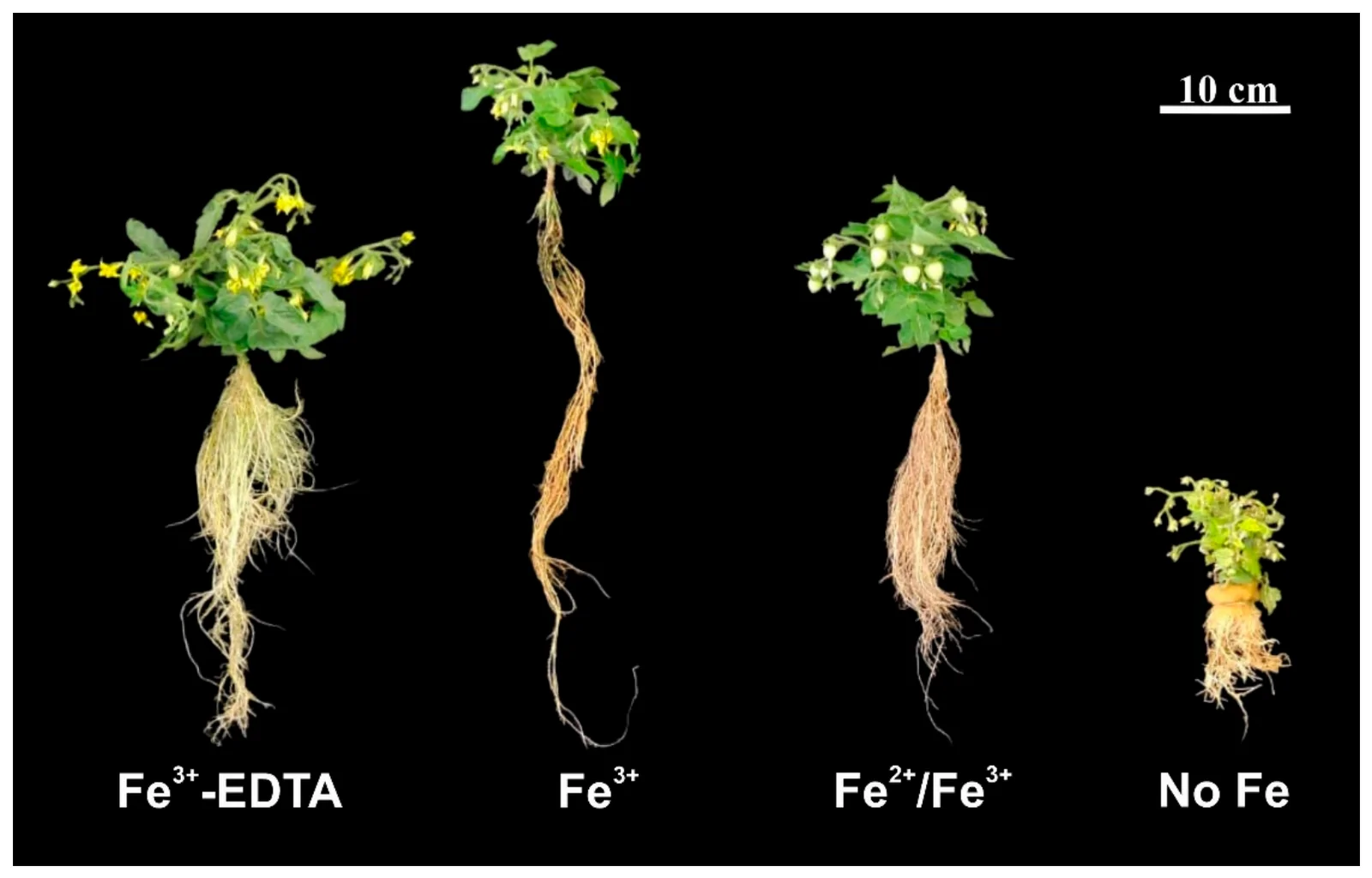
Plants grown in the different treatments: From left to right: Control (Fe^3+^-EDTA); 125 mg L^−1^ of maghemite (Fe^3+^); 125 mg L^−1^ of magnetite (Fe^2+^/Fe^3+^); Iron-free treatment (No Fe).

The dry mass partitioning in plants cultivated with maghemite was higher for leaves and flowers, compared to plants cultivated with magnetite, which showed lower dry mass distribution for the roots (Figure 8 and Figure 9). Maghemite also resulted in greater partitioning to reproductive organs, approximately 1.3 times larger than in plants cultivated with Fe^3+^-EDTA (control).

### 2.6. Heatmap

**Figure 10:**
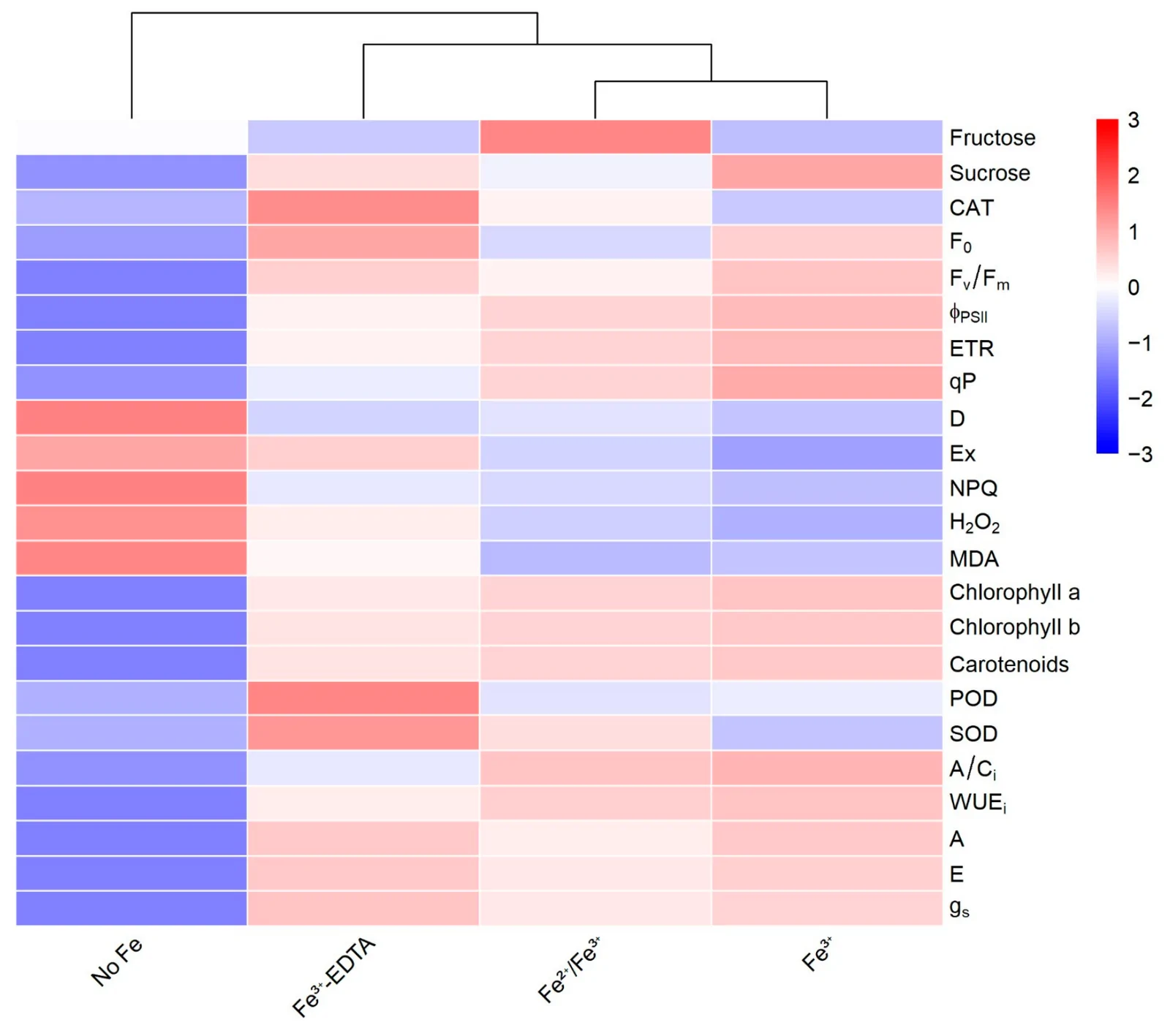
Heatmap. Maximum potential quantum efficiency of photosystem II (Fv/Fm), effective quantum yield of photosystem II (ΦPSII), non-photochemical quenching (NPQ), heat dissipation in the antenna complex (D), energy not dissipated and not used in the photochemical phase (Ex), transpiration rate (*E*), stomatal conductance (*g_s_*), carbon assimilation rate (*A*), instantaneous water use efficiency (WUEi), instantaneous carboxylation efficiency (*A/C_i_*) chlorophyll *a* and *b* and carotenoids, lipid peroxidation (MDA), leaf concentration of hydrogen peroxide (H_2_O_2_), activity of superoxide dismutase (SOD), catalase (CAT), peroxidase (POD), fructose, and sucrose in the Micro-Tom tomato grown in the different treatments. Positive control (Fe^3+^-EDTA); 125 mg L^−1^ of maghemite (Fe^3+^); 125 mg L^−1^ magnetite (Fe^2+^/Fe^3+^); negative control (No Fe).

In the heatmap, the formation of three groups can be observed: a cluster containing treatments with nanoparticles, a second cluster with the Fe-EDTA treatment and a third cluster with the treatment without Fe. Plants with maghemite supply showed a positive correlation for sucrose, Fv/Fm, ΦPSII, qP, *A*, WUEi, *A/C_i_*, Chlorophyll *a* and *b* and Carotenoids, while the treatment without iron showed a high positive correlation for NPQ, D, Ex, H_2_O_2_ and MDA (Figure 10).

**Figure 11:**
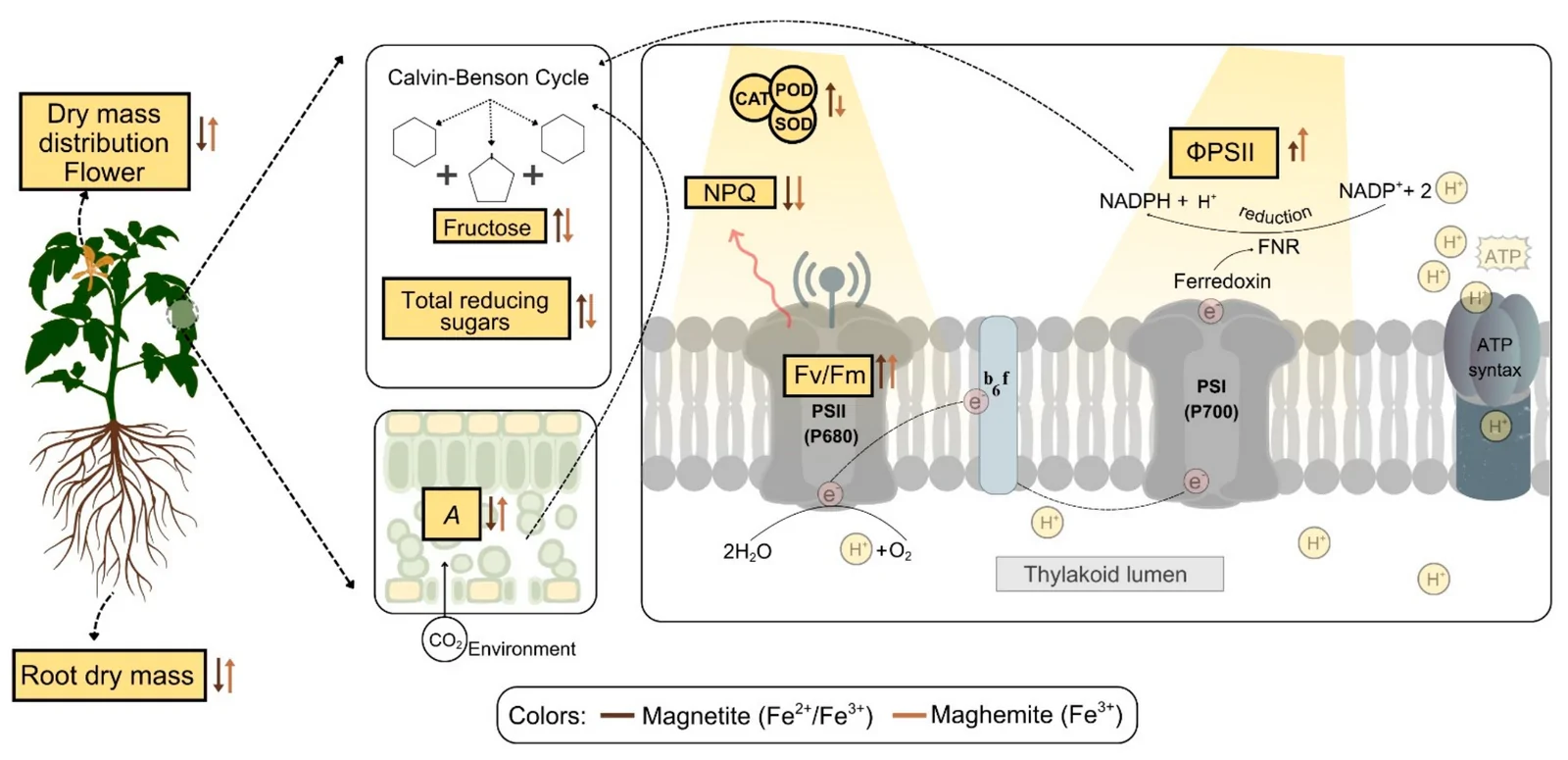
Diagram highlighting the main results observed in Micro-Tom tomato plants grown with maghemite (Fe^3+^) and magnetite (Fe^2+^/Fe^3+^). Maximum potential quantum efficiency of photosystem II (Fv/Fm), effective quantum yield of photosystem II (ΦPSII), non-photochemical quenching (NPQ), carbon assimilation rate (*A*), activity of superoxide dismutase (SOD), catalase (CAT), and peroxidase (POD), fructose and total reducing sugars, root dry mass, and dry mass distribution to the flower. Larger arrows indicate higher values. Dotted arrows indicate metabolic processes.

The following diagram highlights the main observed results, as well as the relationships within plant metabolism and the differences associated with the application of maghemite and magnetite nanoparticles in Micro-Tom tomato (*Solanum lycopersicum*) (Figure 11).

## 3. Discussion

Plants cultivated with maghemite NPs showed higher photosystem stability due to lower energy accumulation in PSII (Ex) and higher effective quantum yield of PSII (ΦPSII). These plants with nanoparticles did not differ from the control (Fe^3+^-EDTA), since the photochemical energy used in the efficiency of PS II (ΦPSII, ETR) was higher and showed equal assimilation. Thus, plants treated with maghemite NPs, which showed a higher effective quantum yield of PSII (ΦPSII) not used for carbon reduction, may have directed part of the energy to alternative electron drains, such as a non-enzymatic defense system [31], since these plants did not present oxidative stress. In *Arabidopsis thaliana*, exposure to 100 mg L^−1^ of zero-valence iron nanoparticles induced stomata opening and increased leaf area, which may contribute to greater light uptake and photosynthetic activity [17]. This condition was not observed in the present study, when 125 mg L^−1^ magnetite or 125 mg L^−1^ maghemite were provided, and it did not lead to an increase in stomatal conductance, transpiration rate, and instantaneous efficiency of water use relative to the control. These results suggest that the physiological effects of iron oxide nanoparticles on plants depend on their class and concentration.

The observation that the photosynthetic pigments were not different in plants treated with iron nanoparticles and EDTA iron confirms the availability and presence of iron in plants. This observation may be supported by the occurrence of the Fenton reaction in the rhizosphere, which involves the interaction of iron with H_2_O_2_, making iron ions available for root uptake [29].

Accumulation of reducing sugars and reduction in MDA and H_2_O_2_ concentrations in Micro-Tom tomatoes grown with magnetite suggests activation of the enzymatic antioxidant system, evidenced by the increase in SOD and POD activities, effective in reducing oxidative stressors. Similar results were observed in other species cultivated under water deficit, which showed increased glucose and fructose in the leaves and lower concentrations of MDA and H_2_O_2_. Plants that showed sugar accumulation resulted in higher stress tolerance [32], which entails the activation of antioxidant enzymes. Thus, potential stress in plants cultivated with magnetite NPs, caused by the increased availability of Fe^2+^, may have led to higher iron uptake and toxicity [20].

The higher carbon assimilation in ‘Micro-Tom’ tomato plants grown with maghemite, combined with lower reducing sugar accumulation in the leaves, suggests the translocation of sugars to sink tissues, such as the roots, which exhibited greater dry mass. According to literature data, sugar distribution within plant tissues may be linked to signaling mechanisms triggered by the slow release of iron ions; this maintains optimal iron levels without inducing phytotoxicity, thereby promoting plant growth [33]. Such growth may be associated with sucrose, a sugar that facilitates cell expansion, consistent with findings reported in the literature [32]. These results agree with those of Yousaf et al. [22], who observed root growth and increased biomass in *Arachis hypogaea* seedlings treated with maghemite nanoparticles, suggesting transport of photoassimilates from the source to the drains. On the other hand, there was lower carbon assimilation and greater accumulation of these reducing sugars in the magnetite treatment, indicating, once again, toxicity due to greater absorption of Fe^2+^ in these plants [20].

Low concentration of sucrose in plants cultivated without iron reinforces the role of this element in organic synthesis, since Fe is a component of the heme group, fundamental in the activation of the activity of cytochrome b6f, as well as of the Fe-S protein, essential for the functioning of ferredoxin, complexes involved in the transport of electrons during the photosynthetic process [34]. Thus, the present study confirms, once again, the essentiality of iron in carbon metabolism.

Low CAT activity in iron-deficient plants may be related to the function of this ion as a cofactor of the heme group of the enzyme [35], which will not exert an oxide-reduction function by transferring electrons from iron to H_2_O_2_ and undergoing its dismutation into two molecules of water and O_2_. Iron deficiency compromises the activation of this enzyme and increases the concentration of H_2_O_2_, which was observed in the treatment without iron. Thus, the plants showed greater signs of stress, with increased lipid peroxidation and the accumulation of MDA. On the other hand, the low activity of CAT in the treatment with maghemite can be explained by the lower accumulation of H_2_O_2_, generating less membrane damage (MDA) in these plants.

Most peroxidase enzymes (POD) have a heme group, a complex formed by iron as the central ion [36]. These enzymes are responsible for neutralizing H_2_O_2_. Fang and Kao [37] have shown that the increase in POD is a result of defense against metals that can cause damage to plants. Thus, the low activities of POD and CAT in the treatment with maghemite suggest that the antioxidant system was not activated, which may have occurred due to the low oxidative stress caused by the lower concentrations of H_2_O_2_ and MDA. The concentration of maghemite NPs used in this study did not cause oxidative stress to the plants.

Magnetite NPs (Fe_3_O_4_) at a concentration of 50 mg kg^−1^, applied to tomato plants, increased fresh shoot biomass by 34% compared to the control. Lower concentrations, such as 25 mg kg^−1^, increased fresh shoot biomass by 26% compared to the control. These plants also showed higher CAT activity than the control [38]. In addition, the use of these NPs at a concentration equal to 50 mg kg^−1^ increased the amount of Fe in the root [38]. The higher absorption of Fe^2+^, when magnetite was applied, suggests that the Ferric Reduction Oxidase 2 (FRO2) transporter remained active in the presence of available Fe^2+^. A similar mechanism may have allowed excess iron uptake in the present study, in which plants were fertilized with 125 mg L^−1^ of magnetite NPs, which may indicate phytotoxicity of the mineral and, once again, explains the high accumulation of reducing sugars in the leaves, low minimum fluorescence (F_0_) and carbon assimilation, lower total and root dry mass and higher antioxidant system activity.

Superoxide dismutase (SOD) enzyme is the first activated defense against oxidative stress. Fe-SOD has iron as a cofactor (Fe-SOD) and is inactivated by H_2_O_2_ at high concentrations [39], and is responsible for protection against oxidative stress in the roots [39,40,41,42]. In this sense, low SOD activity in iron-free plants may have been caused by the absence of the Fe cofactor and high concentrations of H_2_O_2_, inactivating the enzyme. On the other hand, the low activity of SOD in plants cultivated with maghemite suggests its inactivation due to lower oxidative stress. Thus, maghemite can be highlighted as an iron oxide nanoparticle for tomato plant cultivation, among other plant species reported in the literature.

Although the impact of Fe toxicity on plants has not yet been fully understood regarding the molecular cascades involved in this signaling [43], it is known that excess ferrous ions (Fe^2+^) absorbed by the roots can be transported by the xylem to the leaves, causing excess in the leaf tissue. In addition, plants grown with excess Fe show reduced root growth [44,45,46,47], which may have occurred in plants treated with magnetite NPs, as indicated by their lower root dry mass compared to the control and maghemite treatments. Plants cultivated with magnetite also showed reduced photosynthetic efficiency, further supporting the occurrence of physiological responses consistent with Fe-induced stress. Excess Fe reduces photosynthetic rates by disrupting energization of the thylakoid membrane [48,49], as well as speeding up ^1^O_2_ production [50], causing greater oxidative damage by ROS [51], and influencing enzyme activation and protein synthesis [52], which are fundamental for increasing the dry mass of plants.

The literature shows that the supply of Fe^2+^ can impair the development of roots and leaves, resulting in a decrease in root and shoot dry mass, with an increase in leaf lipid peroxidation in several plant species [20,29,30]. In the present study, the supply of magnetite NPs at 125 mg L^−1^ was associated with a physiological response under conditions of Fe excess, characterized by increased accumulation of reducing sugars, lower root dry mass, and increased antioxidant enzyme activity, results consistent with data from the literature [18]. It is important to note that Fe uptake and tissue Fe concentrations were not directly quantified in this study. Therefore, the observed responses do not provide direct evidence of increased Fe^2+^ uptake. Instead, it can be suggested that exposure to magnetite modified Fe availability and/or cellular redox homeostasis, thereby increasing metabolic stress in the plants. The concomitant accumulation of reducing sugars may reflect an imbalance between carbon assimilation and utilization [53], possibly associated with changing carbon export from source leaves or reduced carbon demand under magnetite exposure.

In this study, the plants completed the cycle with 90 days of cultivation. Notably, plants treated with Maghemite showed greater resources in flower formation (Figure 7c). However, this was not observed in the conversion of fruit dry matter (Figure 7b). This result suggests that the physiological advantage associated with maghemite treatment, particularly the maintenance of photosynthetic performance and lower oxidative stress, did not translate into greater final fruit biomass within the experimental period. On the other hand, the investment in the fruits of plants without iron may have occurred as a survival strategy of the species under stress conditions, potentially reflecting an attempt to prioritize reproductive development under nutrient limitation [54]. Therefore, these results indicate that the effects of iron oxide nanoparticles extend beyond iron nutrition itself, involving the coordination of photosynthetic activity, redox homeostasis, carbon metabolism, and biomass partitioning. It is important to highlight that the contrasting responses to magnetite and maghemite suggest that the physiological effects of Fe-based nanoparticles depend not only on nanoparticle concentration but also on the type of nanoparticle, as this determines the availability of divalent or trivalent ions. Maghemite treatment resulted in a stronger positive correlation with sucrose, Fv/Fm, *A*, ΦPSII, Chl *a*, and carotenoids; this suggests that the nanoparticle facilitated the recovery of the photochemical apparatus and was associated with lower antioxidant enzyme activity, enabling the plants to exhibit leaf sugar patterns and total dry mass accumulation comparable to those of the Fe-EDTA treatment (control).

## 4. Materials and Methods

### 4.1. Plant Species and Cultivation

Seeds of Micro-Tom tomato (*Solanum lycopersicum*), wild type, were sown until the development of the first definitive leaves, at 30 days. Subsequently, the plants were grown in nutrient solution, with and without iron-EDTA supply, for 30 days until signs of iron deficiency appeared, prior to being transferred to the treatments; at that stage, the iron-deficient plants exhibited Fv/Fm values close to 0.65. Thus, the plants were grown in nutrient solution n° 2 from Hoagland and Arnon (Table S1) [55] with or without iron or its supply through nanoparticles. Plants cultivated with iron-EDTA for 30 days continued with that treatment. Meanwhile, those cultivated without iron-EDTA that showed signs of deficiency were assigned to the other three treatments: without iron and with nanoparticles. The treatments were designated: control (supply of Fe^3+^-EDTA); no supply of iron; 125 mg L^−1^ of maghemite (Fe^3+^) and 125 mg L^−1^ of magnetite (Fe^3+^/Fe^2+^). The nutrient solution in the different treatments was replaced every 14 days to maintain electrical conductivity (1.25 ± 0.3 mS m^−2^) and pH (5.5–6.5). The experiment was carried out in a greenhouse, with a temperature of 26.0 ± 4 °C, humidity of 31.0 ± 9% and light of 621.3 ± 300 μmol^−2^ m^−2^ s^−1^, and the evaluations were carried out on the 14th, 29th and 40th days after the application of the treatments.

### 4.2. Preparation and Functionalization of Iron Oxide Nanoparticles

Magnetite nanoparticles (Fe_3_O_4_) were synthesized and functionalized according to [23]. Maghemite NPs (γ-Fe_2_O_3_) were obtained by resuspending the magnetite precipitate (Fe3O4) in 50 mL of ultrapure water and maintaining it under agitation at 70 °C, with gradual addition of dilute hydrogen peroxide solution (H_2_O_2_ 10% *v*/*v*) (0.5 to 1.0 mL per cycle, during sonication at 20 kHz). The stabilization of the reddish-brown color was monitored as a qualitative (macrovisual) indicator of γ-Fe_2_O_3_ phase formation. Then, the suspension was kept under agitation for 30 min and cooled to room temperature, and the powder was recovered by centrifugation at 5000 rpm for 10 min, with the nanoparticles washed with ultrapure water up to pH 7.0, and later with absolute ethanol to remove aqueous residues. Superficial functionalization of maghemite (γ-Fe_2_O_3_) was performed immediately after oxidation. Maghemite NPs were resuspended in 20 mL of ultrapure water and sonicated for 10 min to promote deagglomeration. Then, a sodium citrate solution (Na_3_C_6_H_5_O_7_ (P.A, Dinâmica^®^, Indaiatuba, Brazil), 0.20 g dissolved in 5 mL of ultrapure water) was added under vigorous stirring. The suspension was maintained at 80 °C for 30 min, favoring adsorption of citrate^−3^ ions to the surface of the nanoparticles. Then, NPs were recovered by centrifugation at 5000 rpm for 10 min, washed three times with ultrapure water and twice with absolute ethanol (P.A, Dinâmica^®^, Indaiatuba, Brazil), and then dried in a desiccator under vacuum (≤50 mbar) at room temperature for 48 h. The final material was stored in hermetically sealed flasks under N_2_ atmosphere until the moment of use. The characterization of iron oxide nanoparticles was performed using dynamic light scattering (DLS), transmission electron microscopy (TEM), and FTIR spectroscopy; the results are presented in Supplementary Material Figures S1 and S2.

### 4.3. Variables Evaluated

#### 4.3.1. Chlorophyll a Fluorescence and Gas Exchange

The evaluations were carried out between 9 and 11 a.m. on the second or third expanded sheet. Fluorescence and gas exchange were evaluated with an infrared gas analyzer (IRGA, GSF 3000, Walz, Effeltrich, Germany) with a saturation irradiance of 300 μmol m^−2^ s^−1^, coupled to a light fluorometer (LED-ARRAY/PAM-Module 3055-FL, Walz, Effeltrich, Germany). The leaves were adapted to the dark for 30 min, and then a light pulse equal to 4500 μmol m^−2^ s^−1^ was applied to determine the maximum (Fm) and minimum (F0) fluorescence adapted to the dark [56,57].

Fluorescence variables were calculated in maximum potential quantum efficiency of photosystem II (Fv/Fm = (Fm − Fo)/Fm), potential quantum efficiency of PSII (Fv′/Fm′ = (Fm′ − Fo′)/Fm′), electron transport rate (ETR), effective quantum yield of PSII (ΦPSII = ΔF/Fm′), photochemical quenching (qP), non-photochemical quenching (NPQ = Fm − Fm′/Fm′), heat dissipation in the antenna complex (D = 1 − (Fm′ − Fo′)/Fm′)) and excess energy not dissipated or used in the photochemical phase (Ex = 1 − qP × (Fv′/Fm′)) [56].

The gas exchange variables were transpiration rate (*E*, mmol m^−2^ s^−1^), stomatal conductance (*gs*, mmol m^−2^ s^−1^), net carbon assimilation (*A*, μmol m^−2^ s^−1^) and internal CO_2_ concentration (*Ci*, μmol mol^−1^). Instantaneous water use efficiency (WUEi, μmol CO_2_ mmol H_2_O^−1^) was determined as the ratio of assimilated liquid CO_2_ to transpiration. Instantaneous carboxylation efficiency (*A/Ci*, μmol m^−2^ s^−1^ Pa^−1^) was calculated as the ratio between *A* and *Ci* [57].

#### 4.3.2. Photosynthetic Pigments

Leaves ground with liquid nitrogen (0.045 ± 5 g) were macerated with 3 mL of acetone solution and 0.2 M Tris-HCl (P.A, Dinâmica^®^, Indaiatuba, Brazil), pH 7.8 and kept in the freezer for 1 h. After this period, the samples were centrifuged at 5000× *g* for 5 min, and the supernatants were collected for reading in a spectrophotometer at absorbance at 537 nm (anthocyanin), 470 nm (carotenoids), 663 nm (chlorophyll *a*) and 647 nm (chlorophyll *b*). The calculations and transformations were carried out [58].

#### 4.3.3. Dry Mass

Plant material was separated into root, stem, leaf, flower and fruit and dried in an oven with forced air ventilation at 40 °C until constant dry mass was obtained. Then, weighing was carried out on a precision analytical scale 0.0001 g.

The percentage of dry mass in the different plant organs was calculated based on the dry mass of root, stem, leaf, flower and fruit in relation to the total dry mass.

#### 4.3.4. Soluble Carbohydrate Profile

The total soluble sugars were extracted with 80% ethanol (HPLC standard, Sigma-Aldrich^®^, St. Louis, MO, USA), as shown in [59]. The determination of the profile and quantification of soluble sugars was performed by high-performance ion chromatography (Dionex ICS-5000+). The samples and patterns were filtered through 0.22 μm filters and analyzed in a chromatography system equipped with a quaternary pump, autosampler, DCS-5000 electrochemical detector (Thermo Fisher^®^ Waltham, MA, USA), Dionex CarboPac PA100 (Thermo Fisher^®^ Waltham, MA, USA), gold working electrode and Ag/AgCl reference electrode. The eluent phases A, B and C were, respectively, 640 mM sodium hydroxide (HPLC standard, Sigma-Aldrich^®^, St. Louis, MO, USA), 0.5M sodium acetate (HPLC standard, Sigma-Aldrich^®^, St. Louis, MO, USA) and ultrapure water, with a flow rate of 0.7 mL min^−1^ for 35 min [60].

#### 4.3.5. Hydrogen Peroxide Concentration

For the analysis of hydrogen peroxide, 100 mg of frozen and ground fresh leaves were used. Extraction was conducted with 0.1% trichloroacetic acid (TCA, P.A, Dinâmica^®^, Indaiatuba, Brazil) and centrifugation at 12,000× *g* for 15 min at 4 °C. Supernatants were collected and mixed with 0.1 M phosphate buffer (pH 7) and 1 M potassium iodide (KI, P.A, Dinâmica^®^, Indaiatuba, Brazil), with reaction in the dark for 1 h and subsequent absorbance reading in a spectrophotometer at 390 nm. The results were expressed in μmol H_2_O_2_ g^−1^ of fresh mass [61].

#### 4.3.6. Lipid Peroxidation

Lipid peroxidation was performed according to [62]. Thiobarbituric acid (TBA, P.A, Dinâmica^®^, Indaiatuba, Brazil) and trichloroacetic acid (TCA, P.A, Dinâmica^®^, Indaiatuba, Brazil) were used to determine the concentration of malondialdehyde in 100 mg of leaves collected with liquid nitrogen and maintained in a freezer at −20 °C. The readings were performed at 560 and 600 nm for the determination of malondialdehyde, and the results were expressed in nmol of malondialdehyde (MDA) g^−1^ of fresh mass.

#### 4.3.7. Antioxidant Enzymes

The extraction of antioxidant enzymes and total soluble proteins was performed according to [63]. The extract obtained was separated into microtubes and stored at −20 °C for further determination. The activities of the enzymes: superoxide dismutase (SOD, EC 1.15.1.1) [64], Total Peroxidase Pool (POD, EC 1.11.1.7) [64], and catalase (CAT, EC: 1.11.1.6) [65] were determined. The quantification of total proteins was performed according to [66].

### 4.4. Statistical Analysis

The analyses were performed using the R software (version 4.4.2, 2024). Gas exchange and chlorophyll *a* fluorescence were evaluated according to a generalized linear mixed model, considering the time of data collection as a random effect. For the other variables, generalized linear models without random effects were adjusted (Tables S2 and S3). Significance was assessed via analysis of variance (ANOVA, *p* < 0.05) between the complete model and the null model for each variable. A heatmap was generated. The data were auto-scaled prior to analysis, and hierarchical clustering was performed based on Euclidean distance and Ward’s method.

## 5. Conclusions

Due to their slow-release properties, the application of nanoparticles of iron oxide (magnetite and maghemite) could minimize iron losses through leaching while providing sufficient iron to reverse nutrient deficiency and restore photochemical function. However, magnetite supply induced signs of phytotoxicity, including decreased root dry mass, accumulation of reducing sugars, and increased antioxidant enzyme activity. In contrast, maghemite promoted greater allocation of photoassimilates to the flowers.

## Data Availability

This published paper includes all data produced or analyzed during this project.

## Supplementary Materials

The following supporting information can be downloaded at: https://www.mdpi.com/article/10.3390/plants15193033/s1, Table S1: Composition of Hoagland & Arnon (1950) nutrient solution N° 2. All salts are analytical grade (P.A.) and from the Dinâmica® brand.; Table S2. Model structure and model selection for all response variables. Model family indicates the distribution assumed for the response variable; Link indicates the link function used to relate the expected response to the linear predictor. AIC represents the Akaike Information Criterion and ΔAIC is the difference between the AIC of each candidate model and the model with the lowest AIC.; Table S3. Model assumptions and residual diagnostics for all response variables. KS p represents the p-value of the Kolmogorov-Smirnov test for the uniformity of simulated residuals; Dispersion p represents the p-value of the DHARMa test for dispersion; Outlier p represents the P of the DHARMa outlier test; Homogeneity p represents the p-value of the test for homogeneity of residual variance among treatment groups. If p < 0.05, we indicated by *; Figure S1. Bare and citrate-coated iron oxides NPs characterization: (A) Dynamic Light Scattering (DLS), (B) Representative transmission electron microscope (TEM) images of NPs, and (C) Tabulated data summarizes the mean hydrodynamic diameters (±standard deviation (S.D.)), nanoparticle polydispersity (PDI), and nanoparticle surface charges in terms of zeta potential measurements (±S.D.) of NPs.; Figure S2. FTIR plot for bare and citrate-coated Fe_3_O_4_ and γ-Fe_2_O_3_ nanoparticles [67,68,69,70].
