# Supplementary material for: Iron Availability for Micro-Tom Tomatoes via Nanoparticles: Implications for the Antioxidant System and Carbon Metabolism
Source: Plants (Basel). 2026 Oct 4;15(19):3033. doi: 10.3390/plants15193033 (PMC13666591; doi:10.3390/plants15193033)
Supplement: Supplementary file 1 [file plants-15-03033-s001.zip › plants-4559049-supplementary.pdf]

Supplementary material

Iron availability for Micro-Tom tomatoes via nanoparticles: implications on the antioxidant system and carbon metabolism

Rafaela Hemily Cirilo[1], João Pedro Sampaio Gama[1], Felipe Girotto Campos*[1], Carla dos Santos Riccardi[2], and Carmen Sílvia Fernandes Boaro[1]

Table S1: Composition of Hoagland & Arnon (1950) nutrient solution N° 2. All salts are analytical grade (P.A.) and from the Dinâmica® brand.

	Stock solution (g L^{-1})	Nutrition solution 50% (ml L^{-1})
**Macronutrients**		
(M) $NH_4H_2PO_4$	115.03	0.50
(M) KNO_3	101.10	2.50
(M) $Ca(NO_3)_2$ .4H2O	236.16	2.50
(M) Mg(SO4).7H2O	246.50	1.00
**Micronutrients**		
H_3BO_3	2.86	
$MnCl_2.4H_2O$	1.81	
$ZnSO_4.7H_2O$	0.22	0.50
$CuSO_4.5H_2O$	0.08	
$H_2MoO_4.H_2O$	0.02	
**Iron (2)**		
Bissodic EDTA ($C_{10}H_{14}N_2O_8Na_2.2H_2O$)	26.10	0.50
$FeSO_4.7H_2O$	24.90	

Table S2. Model structure and model selection for all response variables. Model family indicates the distribution assumed for the response variable; Link indicates the link function used to relate the expected response to the linear predictor. AIC represents the Akaike Information Criterion and ΔAIC is the difference between the AIC of each candidate model and the model with the lowest AIC.

**Classification**	**Response variable**	**Model family**	**Link**	**Fixed effects**	**Random effects**	**AIC**	**ΔAIC**
Chlorophyll *a* fluorescence	NPQ	Gamma	log	Treatment	Collect	0.66	0.00
Chlorophyll *a* fluorescence	F0	Gaussian	log	Treatment	Collect	456.80	0.00
Chlorophyll *a* fluorescence	Fv/Fm	Gaussian	log	Treatment	Collect	-144.15	0.00
Chlorophyll *a* fluorescence	ϕPSII	Gaussian	log	Treatment	Collect	-138.24	0.00
Chlorophyll *a* fluorescence	qP	Gaussian	log	Treatment	Collect	-141.67	9.59
Chlorophyll *a* fluorescence	Fv'/Fm'	Gaussian	log	Treatment	Collect	-162.48	0.00
Chlorophyll *a* fluorescence	ETR	Tweedie	log	Treatment	Collect	285.84	3.60
Chlorophyll *a* fluorescence	D	Gaussian	log	Treatment	Collect	-165.42	2.46
Chlorophyll *a* fluorescence	Ex	Gamma	log	Treatment	Collect	-201.22	0.00
Gas exchange	*gs*	Tweedie	log	Treatment	Collect	377.29	0.00
Gas exchange	*WUEi*	Gaussian	log	Treatment	Collect	201.61	0.20
Gas exchange	*A/Ci*	Gamma	log	Treatment	Collect	-161.77	37.09
Gas exchange	*E*	Gamma	log	Treatment	Collect	53.71	0.20
Gas exchange	*A*	Tweedie	log	Treatment	Collect	152.74	0.00
Photosynthetic pigments	Chlorophyll *a*	Gamma	log	Treatment	-	183.74	0.00
Photosynthetic pigments	Chlorophyll *b*	Gamma	log	Treatment	-	138.73	0.00
Photosynthetic pigments	Chl *a*/*b*	Gamma	log	Treatment	-	28.01	0.86
Photosynthetic pigments	Carotenoids	Tweedie	log	Treatment	-	155.69	1.88
Soluble carbohydrate profile	Sacarose	Gamma	log	Treatment	-	248.06	0.00
Soluble carbohydrate profile	Fructose	Tweedie	log	Treatment	-	135.41	0.00
Soluble carbohydrate profile	Total reducing sugars	Gamma	log	Treatment	-	252.96	1.5
Antioxidant system	POD activity	Gamma	log	Treatment	-	-86.84	0.00
Antioxidant system	SOD activity	Tweedie	log	Treatment	-	23.67	0.00
Antioxidant system	CAT activity	Gamma	log	Treatment	-	-47.40	0.00
Antioxidant system	MDA	Gamma	log	Treatment	-	90.10	0.00
Antioxidant system	H2O2	Gamma	log	Treatment	-	148.28	0.00
Dry masses and assimilate partition	Total dry mass	Gamma	log	Treatment	-	35.49	0.00
Dry masses and assimilate partition	Fruit dry mass	Tweedie	log	Treatment	-	15.13	0.00
Dry masses and assimilate partition	Flower dry mass	Gamma	log	Treatment	-	-13.28	0.00
Dry masses and assimilate partition	Leaf dry mass	Gamma	log	Treatment	-	8.66	0.00
Dry masses and assimilate partition	Stem dry mass	Gamma	log	Treatment	-	27.74	0.00
Dry masses and assimilate partition	Root dry mass	Gaussian	log	Treatment	-	-28.95	9.33

Table S3. Model assumptions and residual diagnostics for all response variables. KS p represents the p-value of the Kolmogorov-Smirnov test for the uniformity of simulated residuals; Dispersion p represents the p-value of the DHARMa test for dispersion; Outlier p represents the P of the DHARMa outlier test; Homogeneity p represents the p-value of the test for homogeneity of residual variance among treatment groups. If $p < 0.05$, we indicated by *.

Classification	Response variable	KS p	Dispersion p	Outlier p	Homogeneity p
Chlorophyll *a* fluorescence	NPQ	0.39	0.85	1.00	0.18
Chlorophyll *a* fluorescence	F0	0.96	0.67	1.00	0.78
Chlorophyll *a* fluorescence	Fv/Fm	**0.07***	0.70	0.30	**0.01***
Chlorophyll *a* fluorescence	ϕPSII	0.93	0.62	1.00	0.16
Chlorophyll *a* fluorescence	qP	0.10	0.81	0.32	**0.04***
Chlorophyll *a* fluorescence	Fv'/Fm'	0.45	0.64	0.31	**0.02***
Chlorophyll *a* fluorescence	ETR	0.94	0.75	1.00	0.11
Chlorophyll *a* fluorescence	D	**0.01***	0.62	1.00	0.79
Chlorophyll *a* fluorescence	Ex	0.88	0.39	**0.06***	0.46
Gas exchange	*gs*	0.86	0.72	1.00	0.33
Gas exchange	*WUEi*	0.92	0.90	0.31	0.39
Gas exchange	*A/Ci*	0.98	0.92	1.00	0.01
Gas exchange	*E*	0.46	0.13	1.00	0.14
Gas exchange	*A*	0.82	0.69	1.00	0.34
Photosynthetic pigments	Chlorophyll *a*	0.59	0.68	1.00	0.99
Photosynthetic pigments	Chlorophyll *b*	0.73	0.94	1.00	0.86
Photosynthetic pigments	Chl *a*/*b*	0.95	0.68	1.00	0.07
Photosynthetic pigments	Carotenoids	0.80	0.68	1.00	0.98
Soluble carbohydrate profile	Sacarose	0.72	0.44	1.00	0.12
Soluble carbohydrate profile	Fructose	0.91	0.09	1.00	0.23
Soluble carbohydrate profile	Total reducing sugars	0.66	0.77	1.00	0.24
Antioxidant system	POD activity	1.00	0.97	0.12	0.27
Antioxidant system	SOD activity	0.53	0.81	0.11	0.07
Antioxidant system	CAT activity	0.9	0.28	1.00	**0.03***
Antioxidant system	MDA	1.00	0.63	1.00	0.28
Antioxidant system	H2O2	0.9	0.26	1.00	**0.02***
Dry masses and assimilate partition	Total dry mass	0.60	0.14	1.00	**0.01***
Dry masses and assimilate partition	Fruit dry mass	0.45	0.89	1.00	0.32
Dry masses and assimilate partition	Flower dry mass	0.96	0.38	1.00	0.91
Dry masses and assimilate partition	Leaf dry mass	0.94	0.37	1.00	0.83
Dry masses and assimilate partition	Stem dry mass	0.82	0.29	1.00	0.13
Dry masses and assimilate partition	Root dry mass	0.83	0.70	1.00	0.88

Characterization of bare and citrate-coated iron oxide nanoparticles

Results

Fig. S1 shows the size distribution histograms obtained by dynamic light scattering (A), morphological properties (B) and the tabulated data sumarizes the hydrodynamics diameter (Dh), polydispersity index (PDI) and zeta potential (ζ) (B) of bare and citrate-coated Fe_3O_4 and γ-Fe_2O_3 nanoparticles. It could be observed that dynamic light analysis of the bare (citrate-free) magnetite NPs showed a bimodal size distribution. The bimodal peak analysis indicated two clearly separate regions. A minor population, accounting 2.9 % of scattered intensity, with a Dh of 13.5 ± 1.8 nm, indicating a primary magnetite core typically formed during co-precipitation method. The remaining 97.1% of the signal represented the larger population centred at 226.5 ± 67.7 nm, consistent with the presence of particle clusters (D10/133.5 nm, D50/208.2 nm and D90/306.6 nm; span 0.83) associated with large particle sizes, colloidal instability and aggregation. The same aggregation behavior was verified for bare maghemite NPs. A similar bimodal profile was observed with a minor population (0.8% of intensity) at 7.7 ± 1.1 nm and a dominant population (99.2%) centred at 178.9 ± 79.1 nm (D10/85.7 nm, D50/159.9 nm and D90/275.1 nm; span 1.18). In the citrate-coated Fe_3O_4 and Fe_2O_3 samples, it was observed a decreased hydrodynamic diameter values, reaching 29.32 ± 0.5 nm and 15.33 ± 0.8 nm, respectively. Regarding the polydispersity index, both bare samples exhibited relatively higher PDI values (0.230 ± 0.01 for Fe_3O_4 and 0.224 ± 0.05 for γ Fe_2O_3). It can describe a broader and less uniform size distributions associated with the aggregated conditions. After citrate coating, the PDI decreased to 0.115 ± 0.02 for Fe_3O_4 citrate and 0.104 ± 0.1 for γ Fe_2O_3 citrate, indicating a more homogeneous particle size distribution. This behaviour can be confirmed by zeta potential analysis that shifted from values close to neutrality for the bare nanoparticles (-1.45 ± 0.4 mV and -5.31 ± 0.2 mV, both near their isoelectric point) to considerably more negative values after citrate coating (-53.0 ± 0.2 mV and -64.0 ± 0.3 mV) for citrate-coated Fe_3O_4 and γ Fe_2O_3. This increase in surface charge magnitude confirms the adsorption of citrate carboxylate groups onto the nanoparticle surface, generating a stronger electrostatic repulsion between particles and, together with the reduced PDI, corroborates the improved colloidal stability of the citrate coated systems [67].

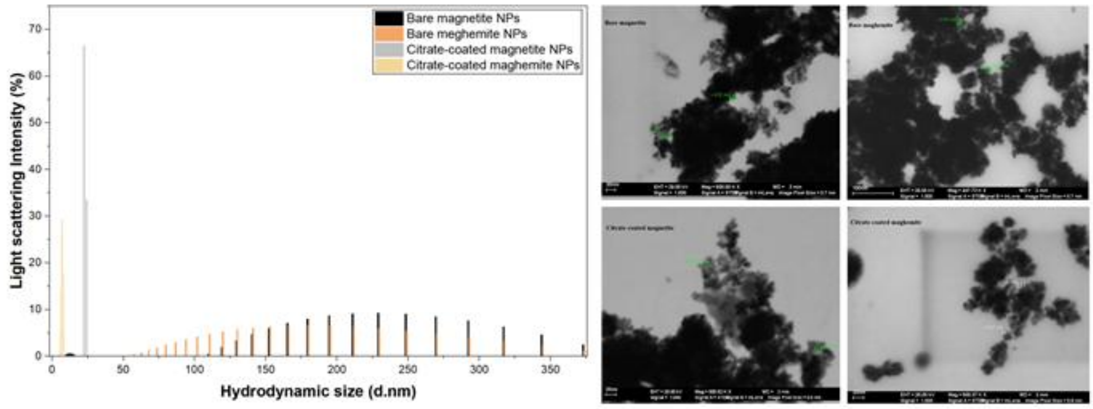

(A) (B)

Samples	Dh (∅) nm	PDI	Zeta potential (ζ) mV
Fe_3O_4	226,5±67,7	0.230±0.01	-1.45±0.4
γ-Fe_2O_3	178.9±79.1	0.224±0.05	-5.31±0.2
Fe_3O_4-citrate	29.32±0.5	0.115±0.02	-53.0±0.2
γ-Fe_2O_3-citrate	15.33±0.8	0.104±0.1	-64.0±0.3

(C)

Figure S1. Bare and citrate-coated iron oxides NPs characterization: (A) Dynamic Light Scattering (DLS), (B) Representative transmission electron microscope (TEM) images of NPs, and (C) Tabulated data summarizes the mean hydrodynamic diameters (±standard deviation (S.D.)), nanoparticle polydispersity (PDI), and nanoparticle surface charges in terms of zeta potential measurements (±S.D.) of NPs.

FTIR spectroscopy (Fig. S2) allowed the identification of absorption bands at 569 cm^{-1}, 1404 cm^{-1}, 1637 cm^{-1}, and 3390 cm^{-1}. The band at 569 cm^{-1} is associated with the stretching vibration of the Fe–O bond, characteristic of the magnetite and maghemite structures. The band at 1400 cm^{-1} can be attributed to the stretching vibrations of carboxyl groups (symmetric and asymmetric), indicating the presence of citrate as a stabilizing agent. The absorption bands at 1637 cm^{-1} and 1404 cm^{-1} can be atributed, respectively, to the asymmetric stretching vibration of the carboxylate functional group (COO-), confirming the presence of chemisorbed citrate íons as a stabilizing agent on the nanoparticle surface. The band at 3390 cm^{-1} is associated with O–H stretching vibrations, related to the adsorption of water molecules on the iron oxide surfaces [67-69].

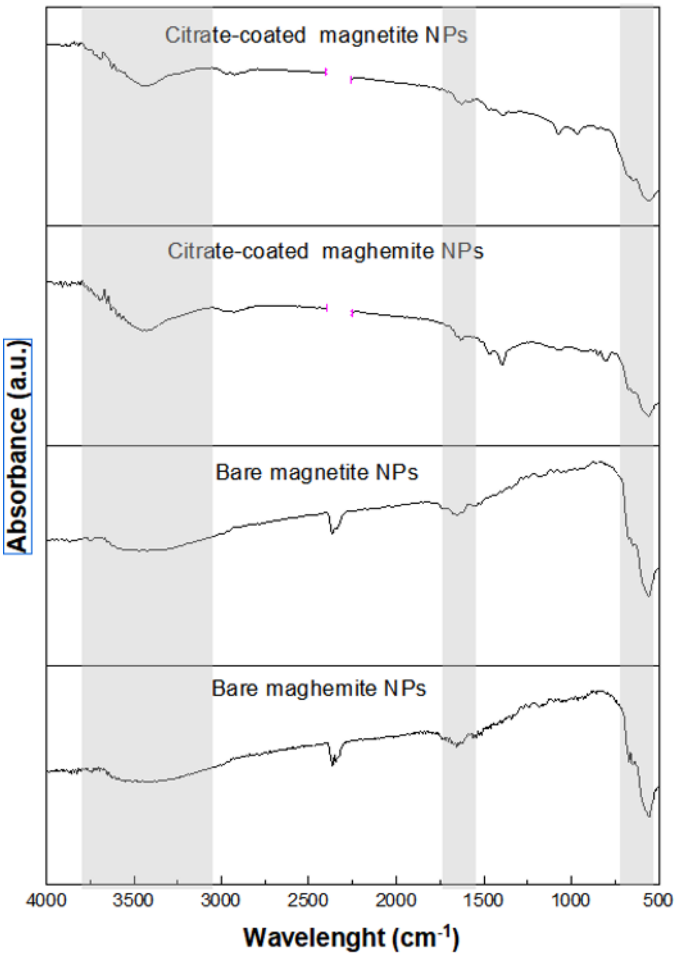

Figure S2. FTIR plot for bare and citrate-coated Fe_3O_4 and γ-Fe_2O_3 nanoparticles

3. Discussion

The preservation of spherical morphology and lattice crystallinity after citrate coating, together with the reduced large-scale agglomeration observed by TEM microscopy, indicates that citrate adsorption acts primarily as a surface stabilizer rather than as a structural modifier of the magnetite or maghemite core [67,70]. This agrees with previous reports that post-synthesis citrate coating does not significantly change the core particle size relative to the uncoated precursor [69], reinforcing that the principal effect of citrate functionalization occurs at the particle–solvent interface rather than within the crystalline lattice itself.

The retention of the Fe–O lattice band after coating confirms that citrate adsorption does not disrupt the underlying iron oxide crystal structure, while the O–H stretching band, present in both bare and coated spectra, reflects surface-adsorbed water and is therefore not itself diagnostic of functionalization. The unambiguous spectroscopic signature of citrate chemisorption is instead the pair of carboxylate (COO^-) bands, absent in the bare oxides and present in every coated sample, together with the downward shift of the asymmetric carboxylate (COO-) band relative to free citric acid.

The bare oxides displayed zeta potentials close to neutrality, with values of −1.45 mV for Fe_3O_4 and −5.31 mV for γ-Fe_2O_3. These magnitudes fall within the rapid flocculation range of the classification scale commonly applied to iron oxide colloids [67,68]. Weak surface charge allows van der Waals and dipolar magnetic forces to dominate particle interactions. This explains the large hydrodynamic diameters observed, 178.9 to 226.5 nm, and the correspondingly broad polydispersity indices of 0,230 para Fe_3O_4 and 0,224 para γ Fe_2O_3. Such values indicate that small primary crystallites aggregated into loosely bound secondary clusters rather than remaining individually dispersed.

Citrate coating shifted both nanoparticle systems into the excellent stability range, exceeding 40 mV in absolute zeta potential. This shift reflects dissociation of citrate carboxylate groups under nearly neutral pH conditions. Unlike previous reports describing citrate coatings that leave particle size largely unchanged [67], the present data reveal citrate acting as an effective deagglomerating agent for the hydrodynamic clusters, without changing the crystalline core size, as identified by TEM microscopy. The hydrodynamic diameter dropped from 226.5 to 29.32 nm for magnetite and from 178.9 to 15.33 nm for maghemite. Polydispersity fell below 0.115 in both cases. These findings suggest that electrostatic repulsion generated by the citrate shell was sufficient not only to prevent further aggregation but also to disperse preexisting clusters into nearly monodisperse colloidal units.

Maghemite showed smaller particle size, greater surface charge magnitude, and lower polydispersity than magnetite after coating. This pattern likely stems from a higher density of accessible surface hydroxyl sites available for citrate coordination on the maghemite lattice. Taken together, citrate functionalization transformed both oxides into compact, highly charged, nearly monodisperse suspensions. Such colloidal behavior favors reproducible interaction with biological interfaces.

In summary, the microscopy, DLS and FTIR data converge na evidence thet citrate functionalization preserves the crystalline core of both magnetite and maghemite while conferring the negative, carboxylate-rich surface chemistry required for colloidal stability and biocompatibility, establishing these citrate-coated nanoparticles as robust, well-characterized platforms for the plant-uptake and phytotoxicity assays addressed in the following sections.

Future work should extend this characterization to the nutrient solution itself, evaluating nanoparticle aggregation kinetics and ion-exchange-driven dissolution under protocols validated for complex exposure media.

4. Materials and Methods

Characterization of bare and citrate-coated iron oxide nanoparticles

The morphology and size of bare and citrate-coated magnetite (Fe_3O_4) and maghemite (γ-Fe_2O_3) nanoparticles (NPs) were characterized by transmission electron microscopy (TEM) on a JEM2100F (JEOL Ltd., Japan) operating at 100 kV. Particle-size distribution from TEM micrographs were computed with ImageJ software and fitted to log-normal curve. The hydrodynamic diameter (Dh), polydispersity index (PDI) and zeta potential (ζ) of the suspension (0.1% w/v) in ultrapure water and phosphate buffer (0.1 M; pH 7.4) for (ζ) were determined at 25 ºC by dynamic light scattering (DLS) using a Zetasizer-type analyzer (Malvern Instruments, Malvern, UK) equipped with a He-Ne laser (633 nm), with each sample measured in triplicate. The results were expressed as mean ± SD. Spectra were collected using an FT-IR spectrometer (PerkinElmer Spectrum Two™-PerkinElmer, Waltham, MA, USA) equipped with a PerkinElmer Universal Attenuated

Total Reflectance (uATR) diamond crystal (single bounce). The spectral data was acquired across a range of 4000 cm−1 to 500 cm−1 at a resolution of 4 cm^{-1}, collecting 24 scans. The spectra were performed for bare and citrate-coated nanoparticles to locate the Fe–O lattice band and the carboxylate (COO^{-}) bands, confirming of citrate chemisorption on the particle surface.

References

[67] Mikelashvili, V.; Kekutia, S.; Markhulia, J.; Saneblidze, L.; Maisuradze, N.; Kriechbaum, M.; Almásy, L. Synthesis and Characterization of Citric Acid-Modified Iron Oxide Nanoparticles Prepared with Electrohydraulic Discharge Treatment. Materials 2023, 16, 746, doi:10.3390/ma16020746.
[68] Dheyab, M.A.; Aziz, A.A.; Jameel, M.S.; Abu Noqta, O.; Mehrdel, B.; Khaniabadi, P.M. Simple Rapid Stabilization Method through Citric Acid Modification for Magnetite Nanoparticles. Sci. Rep. 2020, 10, 10793, doi:10.1038/s41598-020-67869-8.
[69] Silva, A.L.G.; Carvalho, N.V.; Paterno, L.G.; Moura, L.D.; Filomeno, C.L.; de Paula, E.; Báo, S.N. Methylene Blue Associated with Maghemite Nanoparticles Has Antitumor Activity in Breast and Ovarian Carcinoma Cell Lines. Cancer Nanotechnol. 2021, 12, 11, doi:10.1186/s12645-021-00083-x.
[70] Alzoubi, F.; Abu Noqta, O.; AlZoubi, T.; AlJabaly, H.; Alkhateeb, H.; Alqadi, M.; Makhadmeh, G. Exploring the Impact of pH on the Properties of Citric Acid-Coated Iron Oxide Nanoparticles as a High-Performance T2 Contrast Agent for MRI Applications. Results Eng. 2023, 18, 101206, doi:10.1016/j.rineng.2023.101206.
